# Hormonal networks involved in apical hook development in darkness and their response to light

**DOI:** 10.3389/fpls.2014.00052

**Published:** 2014-02-26

**Authors:** Maria A. Mazzella, Jorge J. Casal, Jorge P. Muschietti, Ana R. Fox

**Affiliations:** ^1^Instituto de Investigaciones en Ingeniería Genética y Biología Molecular, “Dr. Héctor Torres” (INGEBI-CONICET)Buenos Aires, Argentina; ^2^Facultad de Agronomía, Instituto de Investigaciones Fisiológicas y Ecológicas Vinculadas a la Agricultura, Universidad de Buenos Aires and CONICETBuenos Aires, Argentina; ^3^Fundación Instituto Leloir, Instituto de Investigaciones Bioquímicas de Buenos Aires-CONICETBuenos Aires, Argentina; ^4^Departamento de Biodiversidad y Biología Experimental, Facultad de Ciencias Exactas y Naturales, Universidad de Buenos AiresBuenos Aires, Argentina

**Keywords:** photoreceptors, gibberellins, ethylene, auxin, apical hook, PIFs, COP1, Arabidopsis

## Abstract

In darkness, the dicot seedlings produce an apical hook as result of differential cell division and extension at opposite sides of the hypocotyl. This hook protects the apical meristem from mechanical damage during seedling emergence from the soil. In darkness, gibberellins act via the DELLA-PIF (PHYTOCHROME INTERACTING FACTORs) pathway, and ethylene acts via the EIN3/EIL1 (ETHYLENE INSENSITIVE 3/EIN3 like 1)-HLS1 (HOOKLESS 1) pathway to control the asymmetric accumulation of auxin required for apical hook formation and maintenance. These core pathways form a network with multiple points of connection. Light perception by phytochromes and cryptochromes reduces the activity of PIFs and (COP1) CONSTITUTIVE PHOTOMORPHOGENIC 1—both required for hook formation in darkness—, lowers the levels of gibberellins, and triggers hook opening as a component of the switch between heterotrophic and photoautotrophic development. Apical hook opening is thus a suitable model to study the convergence of endogenous and exogenous signals on the control of cell division and cell growth.

## Introduction

Endogenous signals such as hormones control the developmental progress during the life cycle of plants. However, as sessile organisms, plants have evolved the ability to dynamically adjust their body form and function in response to the changing environment. These changes tailor the plant phenotype to the prevailing conditions, thus favoring plant survival (Casal et al., 2004). Light is one of the most influential external stimuli controlling plant development. For instance, when seeds germinate buried in the darkness of the soil, the new seedlings follow the skotomorphogenic pattern. In Eudicots, this developmental program is characterized by a fast growing embryonic stem (e.g., hypocotyl in *Arabidopsis thaliana*), the formation of an apical hook structure and the presence of folded cotyledons. Light exposure initiates the transition between skoto- to photomorphogenesis (Kami et al., 2010). The hypocotyl reduces its growth rate, the apical hook opens and the cotyledons unfold while the seedling becomes photosynthetically competent.

In this paper we review the interplay between light signals perceived by photoreceptors (Box 1) and hormonal signals in the control of apical hook development and opening. The main focus is placed on *Arabidopsis thaliana* but information from other species is included to provide a more complete picture.

Box 1 **Light perception and signaling in photomorphogenesis**

**Photoreceptors**

When the shoot emerges from the soil, the light signal that initiates the transition between skoto- and photo-morphogenesis (de-etiolation) is perceived mainly (although no exclusively) by phyA, phyB and cry1. phyA is important for the early steps of this transition, which is completed by phyA itself under dense canopies and by phyB and cry1 in open places (Sellaro et al., 2010; Casal et al., 2013). phyA is activated by radiation between 300 and 780 nm range (Shinomura et al., 1996), but maximally by far-red light (Rausenberger et al., 2011). phyB and cry1 are activated by red and blue light, respectively (Quail et al., 1995; Cashmore et al., 1999) (Figure 4).

**Transcription factors with either positive or negative effects on photomorphogenesis**

PIFs are bHLH transcription factors that bind mainly to the G-box motifs of their target promoters to repress photomorphogenesis (De Lucas et al., 2008; Leivar et al., 2009; Shin et al., 2009; Zhang et al., 2013b). Conversely, HY5 is a b-Zip transcription factor that binds mainly to the G-box motifs of their target promoters to promote photomorphogenesis (Lee et al., 2007; Zhang et al., 2013a). In some cases, PIFs and HY5 may actually compete for the same binding sites (Chen et al., 2013). Light reduces the activity of PIFs and enhances the activity of HY5 (and many other transcription factors with positive action in de-etiolation) to promote photomorphogenesis (Figure 4).

**Signal transduction**

In darkness, phyA and phyB are cytoplasmic homodimers. Light absorption shifts phyA and phyB from the inactive (Pr) to the active (Pfr) form and part of these Pfr pools migrate to the nucleus (Kircher et al., 1999, 2002; Huq et al., 2003), where they bind PIFs (De Lucas et al., 2008; Feng et al., 2008). As a result of this interaction PIFs become phosphorylated and their activity is reduced by a combination of ubiquitination followed by degradation in the 26S proteasome (Al-Sady et al., 2006; Shen et al., 2007) and reduced ability to bind their targets (Park et al., 2012) (Figure 4). cry1 is present in the nucleus and the cytoplasm and light does not significantly change its localization (Wu and Spalding, 2007). In darkness, the E3 ubiquitin-protein ligase COP1 forms a complex with SPA1 and the CUL4-DDB1 E3 ligase core (Lau and Deng, 2012). The multimeric CUL4-DDB1-COP1-SPA1 complex binds ubiquitin to HY5 (and to other transcription factors that promote photomorphogenesis), which becomes targeted to degradation in the 26S proteasome. In the light, the active nuclear pools of cry1, phyA and phyB interact with COP1 (Wang et al., 2001; Yi and Deng, 2005; Liu et al., 2011) and reduce COP1-dependent degradation of transcription factors by a combination of disaggregation of the COP1-SPA1 complex (demonstrated for cry1, Lian et al., 2011; Liu et al., 2011) and translocation of COP1 to the cytosol (von Arnim and Deng, 1994; Osterlund et al., 1999). The traslocation of COP1 to the cytoplasm is a fast process that regulates COP1 activity (Pacín et al., 2014), allowing the pool of nuclear HY5 to build up (Osterlund et al., 2000; Pacín et al., 2014).

**Hormones**

The skotomorphogenic pattern requires brassinosteroids (Chory et al., 1991; Li et al., 1996) and gibberellins (Alabadí et al., 2004). Light reduces the levels of gibberellins (Ait-Ali et al., 1999; Achard et al., 2007; Alabadí et al., 2008) by acting on the expression genes involved in their metabolism (O'Neill et al., 2000; Reid et al., 2002; Zhao et al., 2007). In pea this control involves the COP1/HY5 (Weller et al., 2009). The reduction in gibberellins lowers the activity of the GID1 receptor, involved in a complex that targets DELLA to degradation by the ubiquitin-26S proteosome pathway (Ariizumi et al., 2008) (Figure 4). Thus, in the light DELLAs increase their abundance (Achard et al., 2007) and bind PIFs further reducing their activity (De Lucas et al., 2008; Feng et al., 2008). DELLAs also bind to BRASSINAZOLE RESISTANT1 (BZR1), a transcription factor activated by brassinosteroids, and reduce their activity (Bai et al., 2012; Gallego-Bartolome et al., 2012). These changes in hormone signaling reinforce photomorphogenesis.

## The function of the apical hook

**Figure 1:**
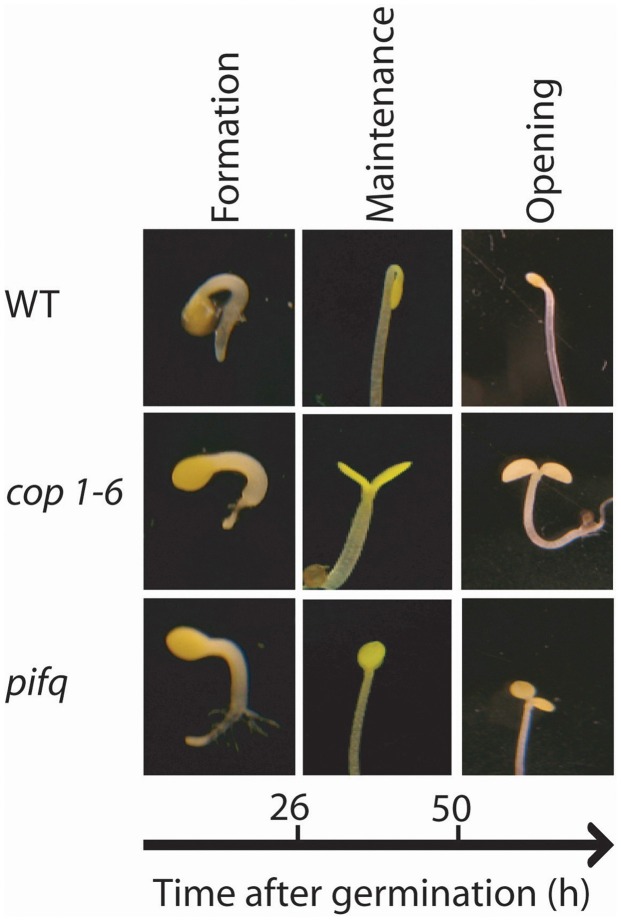
**Apical hook development in dark grown seedlings requires the repressors of photomorphogenesis COP1 and PIFs.** Representative photographs of the apical section of the hypocotyls of WT Col, *cop1-6* and *pifq* mutants during the three phases of hook development. Photographs were taken 24 h after germination for the formation phase, 48 h after germination for the maintenance phase and 84 h after germination for the opening phase.

The apical hook is a transient structure that results from the bending in approximately 180° of the hypocotyl at its apex, below the cotyledons [wild type (WT) in Figure 1]. The apical hook of dark-grown seedlings develops after seed germination to minimize the damage to the shoot apical meristem in its way through the soil to reach the light. The opening of this transient structure formed in the dark must be tightly regulated to ensure seedling survival: if the hook opens prematurely before emergence from the soil, the cotyledons and meristem could be damaged; if the hook opens late, seed reserves could be consumed before the cotyledons become fully exposed to light for photosynthesis.

## Cell growth during apical hook formation, maintenance and opening

Apical hook development takes place in three consecutive phases: formation, maintenance and opening (Raz and Ecker, 1999) (Figure 1). In *A. thaliana* seedlings grown in darkness, the formation phase lasts approximately 26 h after germination while the maintenance phase, where the hypocotyl remains closed, lasts another 25 h (Vandenbussche et al., 2010; Zadnikova et al., 2010; Gallego-Bartolomé et al., 2011). Under prolonged darkness, opening will anyway occur 90–120 h after germination (Vandenbussche et al., 2010; Zadnikova et al., 2010; Gallego-Bartolomé et al., 2011).

**Figure 2:**
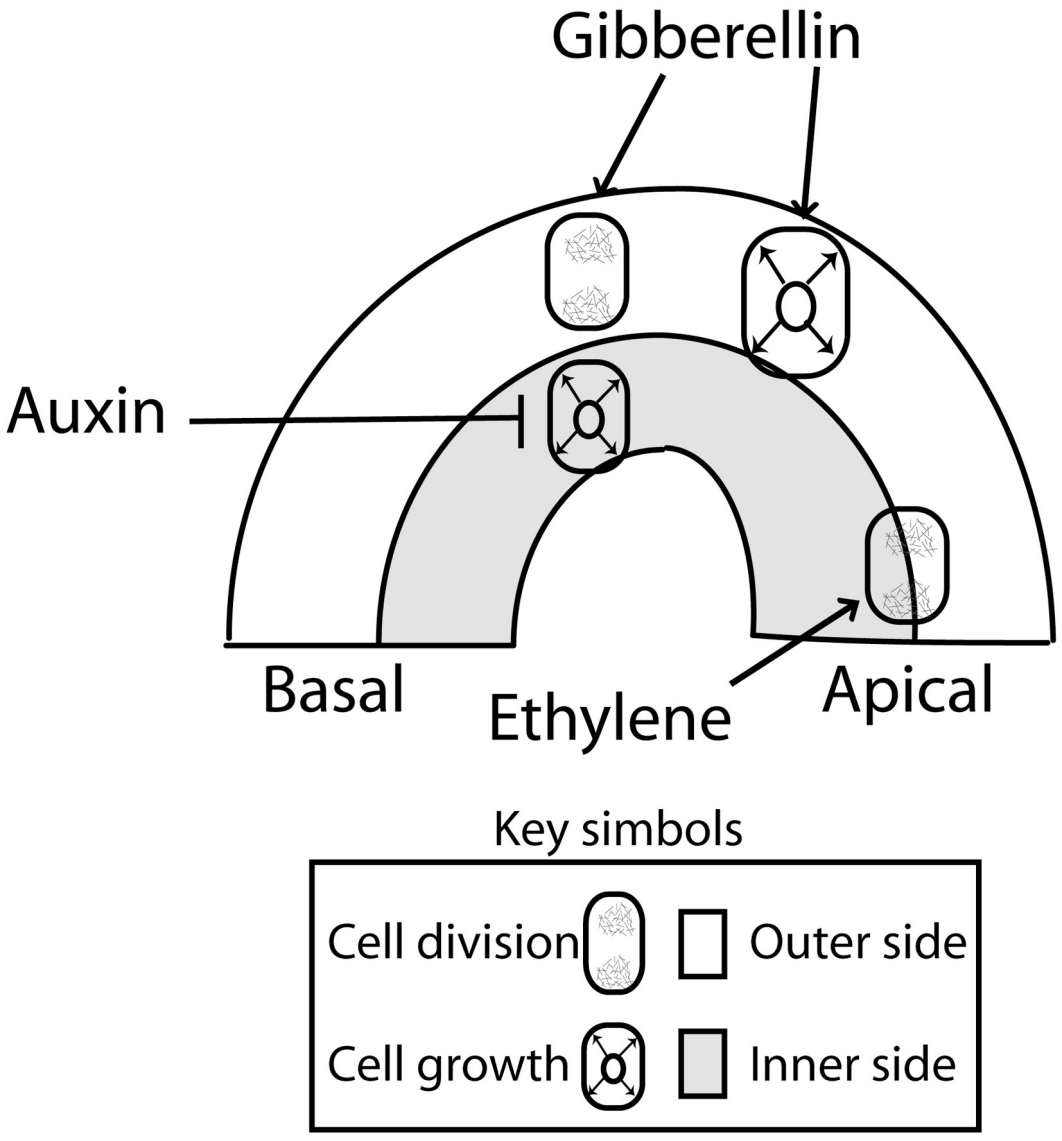
**Primary action of auxin (inhibition of cell expansion at the inner side), gibberellins (promotion of cell division and expansion at the outer side) and ethylene (enhanced cell division at the top) during apical hook development.** There are interactions not represented here, such as the effects of gibberellins and ethylene on the auxin signaling gradient (see Figure 3). Auxin inhibition of cell growth at the inner side of the hook might be mediated by an enhanced ethylene signaling as auxin levels concentrations above a threshold might enhanced ethylene production and signaling contributing to cell growth inhibition (Abel et al., 1995; Raz and Ecker, 1999; Vandenbussche et al., 2010). Arrows: Positive regulation; T-bars: Negative regulation.

The apical hook is formed as the result of localized cell division and asymmetric cell growth at opposite sides of the apical portion of the hypocotyl. To analyze the contribution of cell division and cell growth, it is useful to consider four sections of the apical hook as defined by the apical and basal sides (along the hypocotyl) and the concave (inner) and convex (outer) sides across the hook (Raz and Koornneef, 2001) (Figure 2). When the growth rate of the cells is slower at the inner than at the outer sides of the hypocotyl, the hook is formed and maintained. Conversely, when growth rates at the inner side of the hook exceed those of the outer cells, the hypocotyl straightens and opens (Raz and Ecker, 1999; Vandenbussche and Van Der Straeten, 2004; Vriezen et al., 2004). The main contribution of cell division is evident during the first 24 h of hook development, when more sub-epidermal cells originate at the apical than at the basal portions of the hook (Raz and Koornneef, 2001) (Figure 2). Slightly more cells can be observed at the outer side than at the inner side (Raz and Koornneef, 2001). In sunflower, cell division occurs in the apical hook in coincidence with the high metabolic activity found in that part of the hypocotyl (Kutschera and Niklas, 2013).

## Auxin gradients during hook development

The mechanism underlying differential cell growth in the establishment of hook structure involves a local accumulation auxin at the inner side of the hook (Figure 2). During hook formation and maintenance, the expression of the auxin signaling reporter *DR5:GUS* localizes to the cortex and epidermal cells of the inner side of the hook (Zadnikova et al., 2010; Gallego-Bartolomé et al., 2011). GUS activity driven by *DR5* at the inner side becomes diffuse and fades away during the opening phase. In seedlings treated with an auxin transport inhibitor, no auxin gradient is established, asymmetrical GUS accumulation is blocked and the hook is not formed (Zadnikova et al., 2010). These observations indicate that auxin levels above the optimum would inhibit growth at the inner side of the hook (Figure 2).

**Figure 3:**
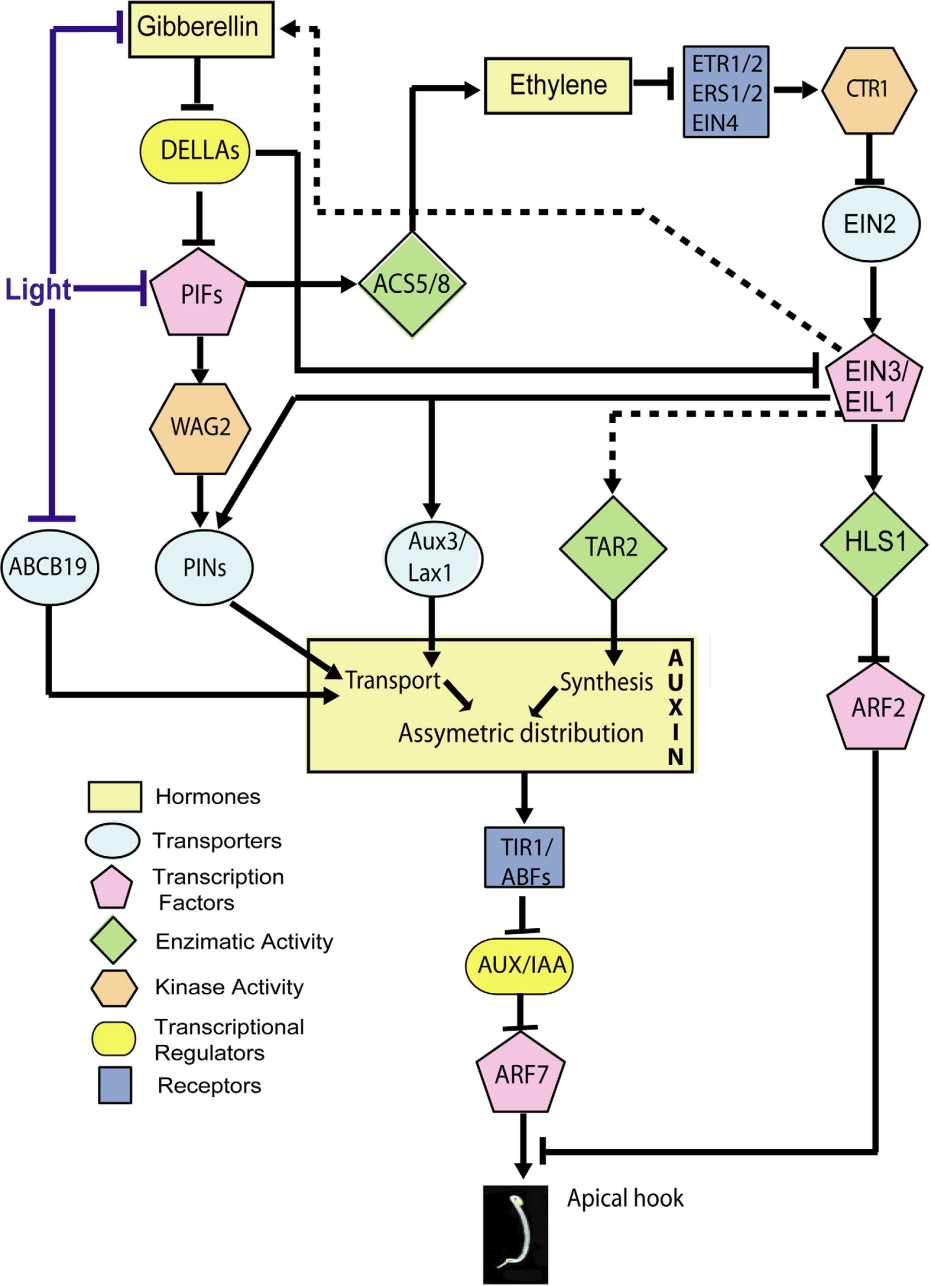
**Hormone signaling network involved in the inhibition of growth at the inner side of the apical hook by auxin.** Established points of action of light during hook opening are indicated. The scheme includes only the components for which specific experimental evidence for a role in apical hook development is available (therefore, for instance, while GID1 should work between Gibberellins and DELLA, this receptor is not included). Lines indicate direct or indirect connections between components. Arrows: Positive regulation; T-bars: Negative regulation. Dotted lines: The expression of *TAR2* is promoted by ethylene and the abundance of gibberellins is promoted by ethylene in an EIN2-dependent manner, but whether these responses depend on EIN3 is not demonstrated.

During apical hook development, the asymmetrical auxin gradient requires normal auxin synthesis (Figure 3). Mutations in the biosynthetic *YUCCA* flavin monooxigenases (Zhao et al., 2001), *SUR1/SUR2* (*SUPERROOT 1/2*) (Boerjan et al., 1995; Delarue et al., 1998) or *TAA1/TAR2* (*TRYPTOPHAN AMINOTRANSFERASE 1-TRYPTOPHAN AMINOTRANSFERASE RELATED 2*) (Stepanova et al., 2008; Vandenbussche et al., 2010) genes impair hook development. The dominant *yuc1-D* mutant contains elevated levels of free auxin and is hookless (Zhao et al., 2001; Vandenbussche et al., 2010). Although auxin synthesis is required for hook development, the auxin gradient is probably not the result of asymmetric auxin synthesis driven by the flavin monooxigenases, because the pattern of GUS activity driven by the *YUC1* promoter is symmetric during hook formation and maintenance (Vandenbussche et al., 2010).

The auxin gradient depends largely on the correct localization of auxin carriers at the plasma membrane. Blocking auxin transport efflux or influx carriers, respectively, by 1-N-naphthylphthalamic acid (NPA) or 1-naphthoxyacetic acid (1-NOA) reduces hook formation (Vandenbussche et al., 2010; Zadnikova et al., 2010). Auxin is transported during hook development by the (PIN) (PIN-FORMED) auxin efflux carriers PIN1, PIN3, PIN4 and PIN7 (Zadnikova et al., 2010), the AUX1/LAX3 (AUXIN-RESISTANT 1/LIKE-AUX 1 3) auxin import carriers and the ABCB19 (ATP-binding cassette (ABC-type) subfamily B number 19 auxin efflux transporter) (Wu et al., 2010) (Figure 3). AUX1 would help to transfer auxin from the cotyledons to the hook; PIN3 carriers would move auxin from the endodermis to the cortex; AUX1, PIN3 and PIN4 from the cortex to the epidermis, and LAX3 would move auxin from the hook toward the roots (Vandenbussche et al., 2010; Zadnikova et al., 2010). *PIN:GUS* expression patterns show differentially spatial and temporal expression for *PIN1*, *PIN3*, *PIN4*, and *PIN7* during apical hook development in darkness (Zadnikova et al., 2010). PIN3 exerts its main role during the formation and maintenance phases while PIN1, PIN4, and PIN7 are important during the maintenance phase and the maintenance to opening transition phases (Zadnikova et al., 2010). The *pin3* (Zadnikova et al., 2010), *aux1* and *lax3* mutants (Vandenbussche et al., 2010) have severe defects in hook development suggesting these transporters have predominant roles in auxin transport in apical hooks. *PIN3* is expressed in the hypocotyl zone where the curvature is maximal, mainly in the outer side of the hook. GFP-ABCB19 is present mostly at the inner side of the hook (Wu et al., 2010). During the opening phase, the enhanced expression at the inner side of the hook is reduced and the auxin asymmetry is lost. Thus, the joint action of these auxin transporters results in the establishment of an auxin gradient toward the inner side of the hook.

Auxin binds to co-receptor complexes formed by TIR1/ABFs F-box receptors (TRANSPORT INHIBITOR RESPONSE 1/AUXIN BINDING F BOX PROTEINS) and AUX/IAA (AUXIN/INDOL-3-ACETIC ACID) proteins (Calderón Villalobos et al., 2012). As result, the AUX/IAA proteins become ubiquitinated and targeted to degradation in the 26S proteosome. AUX/IAA represses ARF (AUXIN RESPONSE FACTOR) transcription factors. Auxin releases ARFs from this inhibition and then ARF activators (such as ARF7 and ARF19) modulate the expression of auxin-responsive genes like the *SAUR* (*SMALL AUXIN UP RNA*) and *GH3* (*GRETCHEN HAGEN 3*) gene families (reviewed by Calderon-Villalobos et al., 2010; Hayashi, 2012). Hook development requires normal auxin perception and signaling. Multiple mutants at receptor loci (Dharmasiri et al., 2005), gain-of-function *iaa1* (Yang et al., 2004), *iaa3*, *iaa12*, *iaa13* (Tian and Reed, 1999; Zadnikova et al., 2010) and *iaa19* mutants (Tatematsu et al., 2004), loss of function *nph4/arf7* and *arf19* mutants (Harper et al., 2000; Zadnikova et al., 2010), lines expressing stabilized versions of the SAUR19-24 proteins (Spartz et al., 2012), and plants overexpressing *SAUR32* (Park et al., 2007) have severe defects in apical hook formation or maintenance (Figure 3). In addition to *SAUR* and *GH3* genes, auxin also induces the expression of some AUX/*IAA* genes. *SAUR32, IAA3:GUS*, *IAA12:GUS* and *IAA13:GUS* reporter lines express GUS activity differentially at the inner side of the hook (Park et al., 2007; Zadnikova et al., 2010).

The *HLS1* (*HOOKLESS 1*) gene encodes a N-acetyltransferase that is required for apical hook formation. The *hls1* mutant fails to form the apical hook due to defects in both differential cell elongation and cell division (the mutant does not express the *CycB1:GUS* marker of mitotic division in the apical portion of the hypocotyl) (Lehman et al., 1996; Raz and Koornneef, 2001). HLS1 is actually required to establish the enhanced auxin-dependent gene expression at the inner side of the apical hook that in turn causes the asymmetric growth (Li et al., 2004).

In addition to the ARFs as activators in auxin-inducible gene expression, there are some ARFs with a negative action. ARF2 is a transcription factor that binds specifically to a DNA sequence present in auxin responsive promoter elements and represses auxin-induced expression (Tiwari et al., 2003) (Figure 3). ARF2 and ARF7 (negative and positive players in auxin signaling, respectively) might bind different promoter regions or might compete each other for the same promoter-binding sites in auxin responsive genes (Ulmasov et al., 1997, 1999; Vernoux et al., 2011). ARF1 and ARF2 function redundantly as repressor of auxin action in apical hook formation (Li et al., 2004). HLS1 reduces the levels of ARF2 (Figure 3). Actually, the *arf2* mutant was identified as a downstream extragenic suppressor of the *hls1* mutation in dark-grown apical hook formation, which partially restores asymmetrical *DR5:GUS* expression (Li et al., 2004). ARF2 protein levels are not affected by auxin (Li et al., 2004); therefore, ARF2 appears to provide an auxin-independent repression of auxin-induced genes (Vernoux et al., 2011).

In summary, an asymmetrical auxin gradient in apical hooks leads to differential cell growth and hook formation and maintenance. The establishment of the auxin gradient requires normal auxin synthesis, transport, perception and signaling. The final result of auxin signaling on hook development would be mediated by the combined actions of activators and repressors ARFs on auxin-induced genes. Together with auxin, gibberellins, ethylene and brassinosteroids contribute to hook formation and are discuss in the following sections.

## Normal hook development requires gibberellins

Gibberellins are important during the initial phase and determine the speed and the degree of hook formation (Gallego-Bartolomé et al., 2011). Seedlings treated with an inhibitor of gibberellins biosynthesis show decreased *DR5:GUS* expression at the inner side of the hook but the deficient hook of the *pin3 pin7* double mutant cannot be restored by gibberellins (Gallego-Bartolomé et al., 2011). These observations indicate that gibberellins act in part by favoring the generation of a normal auxin gradient (Figure 3). In addition, gibberellins appear to enhance apical hook formation more directly, by promoting cell elongation and cell division at the outer side of the hook (Vriezen et al., 2004) (Figure 2). This suggests that the hook is formed by the auxin-mediated inhibition of growth at the inner side and the gibberellin-mediated promotion of growth at the outer side.

Hook formation requires gibberellins to reduce the abundance of DELLAs (Alabadí et al., 2004; Gallego-Bartolomé et al., 2011) (Figure 3). DELLAs are nuclear proteins of the GRAS family (Bolle, 2004), which repress growth. When gibberellins bind the receptor GID1 (GIBBERELIN INSENSITIVE DWARF 1) the latter forms a complex that catalyses ubiquitination of DELLA, leading to their rapid degradation and the release of growth (Ueguchi-Tanaka et al., 2007; Willige et al., 2007; Ariizumi et al., 2008). Five DELLA genes are present in Arabidopsis (*GAI*, *RGA*, *RGL1*, *RGL2*, and *RGL3*) (Peng et al., 1997; Silverstone et al., 1998). The *rga* and *gai* loss of function mutations suppress the hookless phenotype of 3-d-old etiolated gibberellin deficient *ga1-3* mutants (Achard et al., 2003). The quintuple *della* mutant shows a steeper slope of hook angle formation than the WT during the first day after germination, but thereafter it behaves similarly to the WT. These results suggest that gibberellins requirement during apical hook development is important during the formation and maintenance phases (Gallego-Bartolomé et al., 2011).

The quintuple *della* mutant displays closed hooks while the *hls1 della* mutant is completely hookless suggesting that the effects of gibberellins and DELLA depend on HLS1 (An et al., 2012). Gibberellins promote *HLS1* expression (Gallego-Bartolomé et al., 2011; An et al., 2012) and the *gai*-1 mutant allele, that encodes a dominant version of DELLA protein GAI, reduces *HLS1* expression (Gallego-Bartolomé et al., 2011). DELLA proteins directly interact with the DNA binding domain of EIN3/EIL1 (ETHYLENE INSENSITIVE 3/EIN3 LIKE 1), repressing its function (Figure 3). EIN3/EIL1 are transcription factors that interact directly with the *HLS1* promoter to activate its transcription (An et al., 2012) (Figure 3). Thus, gibberellins release EIN3/EIL1 from DELLA inhibition to promote *HLS1* expression (An et al., 2012).

In addition, DELLAs interact directly with PIF3 (PHYTOCHROME INTERACTING FACTOR 3) and PIF4 (see Box 1), interfering with their ability to bind their target gene promoters (De Lucas et al., 2008; Feng et al., 2008) (Figure 3). PIFs are helix-loop-helix transcription factors and at least PIF1, PIF3, and PIF5 promote hook development in darkness (Khanna et al., 2007; Leivar et al., 2008; Gallego-Bartolomé et al., 2011). Gibberellins and at least *PIF1* expression in the endodermis are required for hook formation (Gallego-Bartolomé et al., 2011; Kim et al., 2011). The connection between the gibberellin-DELLA-PIF module and the auxin gradient would be established by WAG2 (a member of the AGC3 kinase subclass family) (Willige et al., 2012). *WAG2* is expressed in a gibberellin-dependent manner at the inner side of the apical hook, and PIF5 binds to a G-box of the *WAG2* promoter increasing its expression (Willige et al., 2012) (Figure 3). WAG2 is an auxin transport-regulatory protein kinase that phosphorylates *in vitro* the central intracellular loop of PIN1, PIN3, PIN4, and PIN7 proteins (Willige et al., 2012) (Figure 3). Four day-old dark-grown *wag2* mutant seedlings display more open hooks than WT seedlings, but 2-d-old dark-grown seedlings are indistinguishable from the WT, suggesting WAG2 is important during apical hook maintenance and the repression of apical hook opening but not during apical hook formation.

So, gibberellins are crucial for apical hook formation. Gibberellins are needed to maintain DELLA levels reduced and thus PIF and EIN3/EIL1 transcription factors are available to increase WAG2 and HLS1 which regulate asymmetric auxin accumulation.

## Ethylene promotes auxin synthesis, transport and signaling at the inner sides of apical hooks

Dark-grown seedlings treated with exogenous ethylene exhibit an exaggerated apical hook, which is one of the components of the classical triple response. In addition, an exaggerated hook is observed in dark-grown dominant mutants that display elevated levels of ethylene, like *ethylene overproducer* mutants (single mutants *eto1, eto2*, and *eto3*) (Guzman and Ecker, 1990; Vogel et al., 1998b; Woeste et al., 1999); and reduced hook curvature is observed in mutants that fail to increase ethylene biosynthesis like *cytokinin insensitive* (single mutants *cin1, cin2, cin3*, and *cin4*) (Vogel et al., 1998a). Furthermore, *eto2* is a dominant mutation of the ethylene biosynthesis *1-aminocyclopropane-1-carboxylate synthase ACS5* gene (Vogel et al., 1998b). Thus, ethylene biosynthesis is important in the regulation of hook curvature. Ethylene has a role during the formation phase and a predominant role in hook maintenance by controlling cell division mainly along the apical-basal parts of the hooks (Raz and Koornneef, 2001) (Figure 2).

Ethylene is perceived by the ETR1, ETR2 (ETHYLENE RESISTANT 1 AND 2), ERS1, ERS2 (ETHYLENE RESPONSE SENSOR 1 AND 2) and EIN4 (ETHYLENE INSENSITIVE 4) receptors (Bleecker, 1999; Schaller and Kieber, 2002). In the absence of ethylene, the receptors are active (Hua and Meyerowitz, 1998) (Figure 3). Dominant mutations in these receptors (like single mutants *etr1-1, ers1-1*) confer ethylene insensitivity and lack of an apical hook, while loss-of-function mutations (like single mutants *etr1-7*, *etr2-3*, *ein4-7*) cause hypersensitivity to ethylene and exaggerated hooks after ethylene treatment (Hua et al., 1998; Sakai et al., 1998; Raz and Ecker, 1999; Qu et al., 2007; Liu and Wen, 2012). These receptors activate CTR1 (CONSTITUTIVE TRIPLE RESPONSE 1) (Clark et al., 1998), which negatively regulates EIN2 (ETHYLENE INSENSITIVE 2) and EIN3/EIL1 (Figure 3). The *ctr1* mutant displays an exaggerated hook phenotype (Knee et al., 2000).

In addition to its effects on cell division, ethylene also acts indirectly by inducing auxin transport and increasing auxin levels at the inner side of the hook (De Grauwe et al., 2005; Zadnikova et al., 2010) via different pathways that target auxin synthesis, auxin transport and auxin-induced gene expression (Figure 3).

Ethylene enhances the expression of the auxin synthesis gene *TAR2* at the inner side of the hook 3 days after germination (Vandenbussche et al., 2010) (Figure 3). In addition, EIN3 binds the promoter of *ASA1 (ALPHA SUBUNIT OF ANTHRANILATE SYNTHASE 1*), the enzyme that catalyzes the first limiting step in tryptophan synthesis (Chang et al., 2013). Auxin accumulation in the root tips is promoted by ethylene through the promotion of *ASA1* expression, and the GUS activity of the transcriptional reporter *ASA1:GUS* is not enhanced by ethylene in an *ein2* mutant (Stepanova et al., 2005). Thus, it is reasonable to speculate that ethylene effects on *TAR2* are mediated by EIN3.

Ethylene enhances the expression of *PIN1*, *PIN3*, *PIN4*, and *PIN7* auxin efflux carriers (Zadnikova et al., 2010) and *AUX1* and *LAX1* auxin influx carriers (Vandenbussche et al., 2010) (Figure 3). The hookless phenotype of the *pin3* mutant seedlings displays reduced sensitivity to ethylene (Zadnikova et al., 2010). NPA treatments almost completely suppress the exaggerated hook phenotype in *ctr1* mutants and *EIN3* overexpressor seedlings (An et al., 2012), suggesting auxin functions downstream of EIN3/EIL1. Also, EIN3 binds *PIN7* and *AUX1* promoters (Chang et al., 2013) (Figure 3). Thus, ethylene would modulate auxin distribution by controlling the expression of auxin transporters through EIN3/EIL1 transcription factors. Modulation of auxin transport in apical hooks is a common event regulated by ethylene and gibberellin actions (Figure 3).

Ethylene promotes *HLS1* expression (Lehman et al., 1996; Du and Kende, 2001; Li et al., 2004; An et al., 2012) (Figure 3) and HLS1 abundance (Li et al., 2004) and also reduces ARF2 abundance (Li et al., 2004). The *hls1* mutant remains hookless even after being treated with ethylene (Li et al., 2004). The loss of phototropic response in the *arf7* mutant can be compensated by ethylene treatment but not that of the *arf7 hls1* double mutant (Harper et al., 2000). This suggests that ethylene would favor the action of additional activating ARFs on auxin signaling by repressing the action of ARF2 via HLS1.

Ethylene also promotes gibberellins synthesis (Vriezen et al., 2004). Treatment with an ethylene precursor enhances the expression of the gibberellin biosynthetic gene *GA1* in apical hooks of 3-d-old dark-grown seedlings (Vriezen et al., 2004) and the expression of a gibberellin responsive promoter fragment *GASA1* at the outer side of the hook (Vriezen et al., 2004) (Figure 3). The promotion of *GA1* expression by ethylene is not observed in the *ein2* mutant (Vriezen et al., 2004). Even though the expression of *GA1* gene by ethylene has not been tested in an *ein3* mutant, from the above results we speculate that gibberellin synthesis might be affected downstream EIN3/EIL1 ethylene regulator.

In addition to the effects of ethylene on gibberellins there is evidence for a reciprocal control. Ethylene production is enhanced in *della* mutants (Gallego-Bartolomé et al., 2011). PIF5 binds a G-box in the promoter of *ACS8* and induces its transcription in a gibberellin and DELLA-dependent manner (Gallego-Bartolomé et al., 2011) (Figure 3).

The previous paragraphs describe the pathways by which ethylene affects the asymmetric auxin-induced responses. Ethylene could also act downstream auxin, which might stimulate ethylene production or ethylene sensitivity at the inner side of the hooks (Raz and Ecker, 1999). In pea epicotyls, the transcript levels of the *PsACO1*, (the isoform of an ACC oxidase) are higher in the inner than in the outer side (Peck et al., 1998). Although this hypothesis has been largely accepted, it is not clear how ethylene would inhibit cell growth at the inner side of the hook.

In summary, normal ethylene production and signaling is necessary for the establishment of the apical hook. Ethylene signaling controls the responses of transcription of several genes through EIN3/EIL1 transcription factors integrating ethylene with gibberellin and auxin in apical hooks.

## Brassinosteroids are required for hook formation

Mutants defective in brassinosteroid synthesis such as *det2* (*deetiolated* 2) lack an apical hook in darkness (Chory et al., 1991). Adding brassinosteroids or inhibitors of brassinosteroid synthesis alters the patterns of auxin response in the hook (De Grauwe et al., 2005) highlighting the function of this hormone during hook formation.

Brassinosteroids enhance the activity of the kinase BIN2 (BR-INSENSITIVE 2) that phosphorylates ARF2 and reduces its activity, leading to the enhanced expression of auxin responsive genes (Vert et al., 2008). Some studies suggest that ethylene could control the apical hook partially by activating brassinosteroid biosynthesis (De Grauwe et al., 2005) or downstream signaling components (Gendron et al., 2008). Analysis of dark-grown seedlings carrying a *CPD:GUS* (CONSTITUTIVE PHOTOMORPHOGENESIS AND DARFWISM) reporter gene which is involved in brassinolide synthesis, shows induced expression of the reporter gene by ethylene in apical hooks. Also, dark-grown *det2* mutant seedlings treated with exogenous ethylene are not able to induce an exaggerated hook suggesting ethylene action requires brassinosteroids (De Grauwe et al., 2005). The double mutant *bri1 bzr1-1D* (*brassinosteroid insensitive 1*; *brassinazole resistant 1*) that is deficient in the perception of brassinosteroids but have activated downstream brassinosteroids responses due to a dominant mutation that stabilizes BZR1, forms and apical hook similar to that of the WT (Gendron et al., 2008). Thus, ethylene might also act regulating brassinosteroid downstream signaling. It has been proposed that brassinosteroids affect auxin distribution through PIN modulation (De Grauwe et al., 2005), however further work is necessary to define the points of regulation of brassinosteroids in the network signaling that leads to apical hook formation.

## Light perceived by phytochromes and cryptochromes induces hook opening

Apical hook opening is one of the responses affected by the switch from skotomophogenic to photomorphogenic development. In most dicotyledonous plants, including *Gossypium hirsutum* L., *Phaseolus vulgaris* L., (Powell and Morgan, 1970) *Pisum sativum* (Britz and Galston, 1982) and *A. thaliana* (Liscum and Hangarter, 1993a), light induces apical hook opening. In some species, like *Solanum Lycopersicum*, characterized by a “hard to split” seed coat, light exaggerates hook formation possible as part of a mechanism that facilitates seed coat removal when seedlings germinate below the soil (Shichijo et al., 2010).

**Figure 4:**
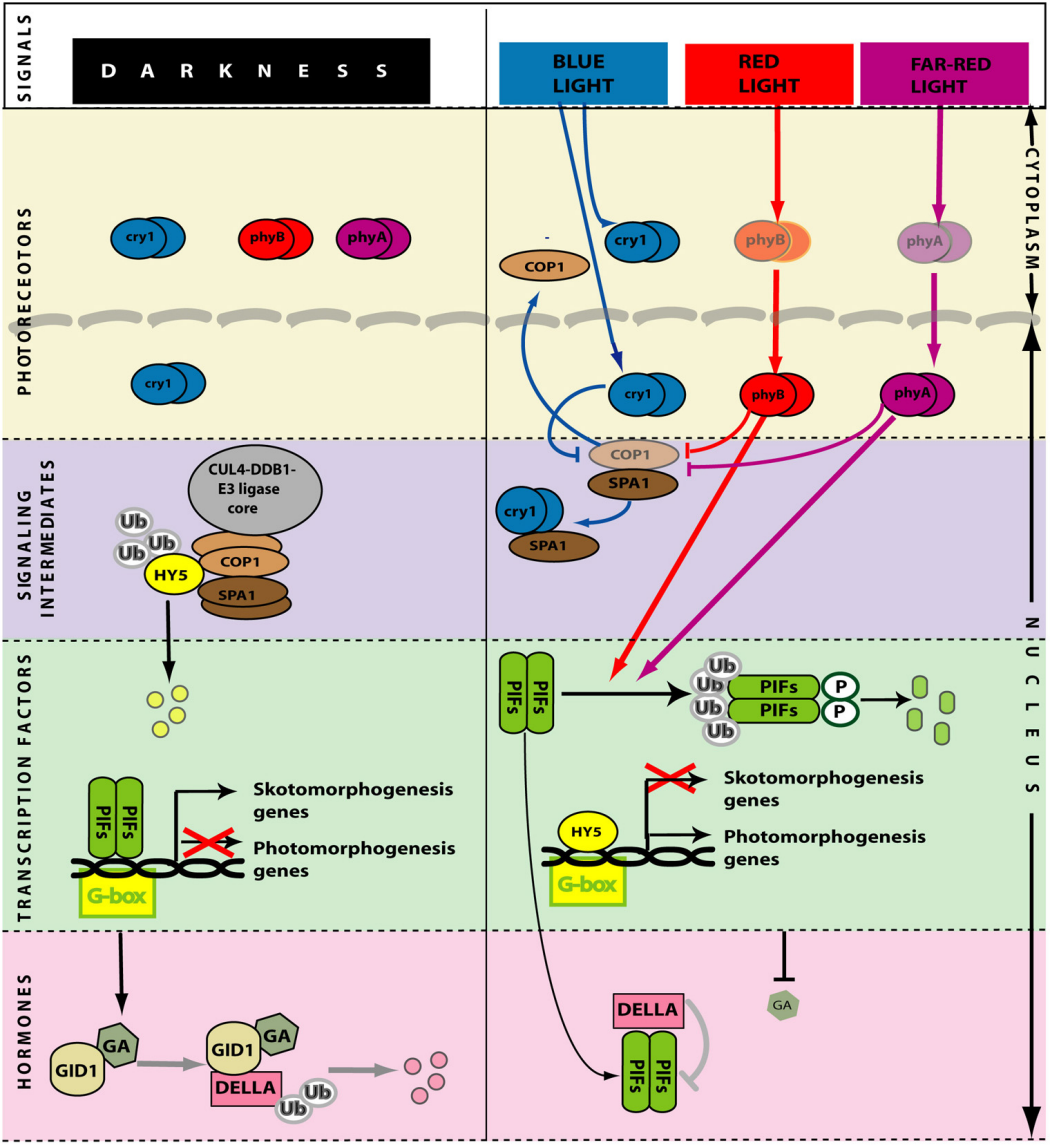
**Simplified model of light perception and signaling during de-etiolation.** In darkness (**left**) the photoreceptors are inactive. PIF transcription factors promote skotomorphogenesis. HY5 and other transcription factors that promote photomorphogenesis are ubiquitinated by CUL4-DDB1^COP1-SPA1^ E3 ligase complex and degraded in the 26S proteasome. High levels of gibberellins induce degradation of DELLA. Light (**right**) activates the photoreceptors. In the nucleus, phytochromes (that migrate from the cytoplasm in their active form) and cryptochromes interact with COP1, reducing its activity and allowing the abundance of HY5 to increase. In the nucleus, phytochromes also reduce the activity of PIFs. Gibberellin levels are reduced, DELLA accumulate and bind PIFs further reducing their activity. GA, gibberellins. Arrows: Positive regulation; T-bars: Negative regulation.

When *A. thaliana* seedlings are exposed to light, the hook structure opens completely within approximately 6 h (Liscum and Hangarter, 1993a; Wang et al., 2009). Blue, far-red and red light induce hook opening via the partially overlapping action of phytochromes and cryptochromes (Liscum and Hangarter, 1993a,b) (see Box 1; Figure 4). In hook opening of *A. thaliana*, red light is the least effective of these colors and its effect is mediated by the redundant actions of phytochrome A (phyA) and phyB (Reed et al., 1994). Far-red light perceived by phyA has a much stronger effect on the apical hook (Liscum and Hangarter, 1993b). Finally, the strong stimulation of hook opening by blue light appears to be mediated by cryptochromes 1 (cry1) in same cases (Liscum and Hangarter, 1993b), but not in others (Wang et al., 2009; Fox et al., 2012). In these cases blue light effects could be mediated by phyA (Casal and Mazzella, 1998; Neff and Chory, 1998; Chun et al., 2001). No function for other phytochromes (phyC, phyD, or phyE) or cry2 has been described for apical hook opening.

In addition to their role under specific wavelengths, photoreceptors are able to affect some processes in darkness downstream of light signals that were perceived at the seed stage, adjusting young seedlings to the environment they will likely have to face (Mazzella et al., 2005). *cry1* mutants grown in darkness on agar containing sucrose display more opened hooks than WT seedlings at late stages of hook development, a phenotype also observed in mutants of the heterotrimeric G *alpha* subunit protein (GPA1) (Fox et al., 2012).

In apical hook cells of dark-grown pea, immunochemically detectable PHYA is distributed diffusely in the cytosol and appears in the nucleus upon exposure to continuous far-red or red light (Hisada et al., 2000) (see Box 1; Figure 4). In 8-d-old, dark-grown soybean seedlings, 1 h far-red light enhances the expression of genes mostly involved in cell division and protein turnover in the hook itself (Li et al., 2011), indicating that the phytochrome signal reaches the nucleus. The translocation of phyA to the nucleus requires FHY1 (FAR RED ELONGATED HYPOCOTYL 1) and FHL1 (FAR RED ELONGATED HYPOCOTYL LIKE 1) (Hiltbrunner et al., 2006; Rosler et al., 2007). The *fhy1* mutant is impaired in apical hook opening under far-red light (Hiltbrunner et al., 2006) suggesting that far-red-induced apical hook opening needs phyA in the nucleus.

Thus, light triggers apical hook opening through the action of photoreceptors phyA phyB and cry1. This process must imply the disruption of the auxin gradient that leads to differentially cell growth. Downstream targets of photoreceptors involved in hook development will be discussed in the next sections.

## Light reduces the activity of PIFs and COP1, which are required for hook development in darkness

Photomorphogenesis is a process repressed in darkness principally by two pathways (Box 1; Figure 4). One involves the E3 ubiquitin ligase COP1 (CONSTITUTIVE PHOTOMORPHOGENIC 1) and the other involves the group of PIF transcription factors (Leivar and Quail, 2011; Lau and Deng, 2012) (see Box 1; Figure 4). The *cop1* mutant (Deng et al., 1992; Alabadí et al., 2008) and the *pif1 pif3 pif4 pif5* quadruple mutant (*pifq*) (Leivar et al., 2008; Shin et al., 2009) have a de-etiolated phenotype when grown in darkness. Both *cop1* and *pifq* are not able to form an apical hook (Figure 1). Thus, COP1 and PIF proteins are indispensable for hook formation. In the presence of light, at least phyA, phyB, and cry1 activities repress COP1 protein while phyA and phyB repress PIF proteins (see Box 1; Figure 4).

## Downstream of PIFs and COP1

Apical hook formation implies the establishment of an auxin signaling maxima and the asymmetric distribution of auxin at the apical hook but this asymmetry has to be compensated during the opening phase. *DR5* expression signals are very strong in the concave side of the dark-grown seedlings, but this asymmetry is greatly reduced or even disappears during the opening phase either in the dark (Zadnikova et al., 2010; Willige et al., 2012) or upon exposure to light (Wu et al., 2010). PIFs have several important points of action leading to the generation of the auxin gradient and hook development in darkness (Figure 3). Therefore, the reduction of PIF activity by phytochromes (Box 1; Figure 4) is predicted to affect the auxin gradient via these pathways. In support of this view, light-induced changes have been documented for some elements that would operate downstream of PIFs in darkness. For instance, *WAG2* transcript levels decrease (Willige et al., 2012) (Figure 3), *HLS1* transcript levels also decrease and ARF2 protein levels increase after light exposure (Li et al., 2004). As expected, ARF2 accumulation was not observed in light exposed *hls1* mutants (Li et al., 2004) (Figure 3). Ethylene sensitivity and/or responses in the apical hook are attenuated by phyA, phyB and cry1 activation (Knee et al., 2000), so phytochrome-mediated modulation might involve the control of *ACS8* expression by PIF5 (Figure 3).

The reduction of PIF3 levels by red light has other consequences. For instance, 1 h after the beginning of red light, low PIF3 activity reduces the expression of the *BBX23* gene (a member of the B-box family of transcription factors). Since BBX23 is a repressor of photomorphogenesis required for hook maintenance in darkness, down-regulation of its expression enhances hook opening (Sentandreu et al., 2011). At later stages, (3–h after the beginning of red light) the reduced PIF3 levels favor the induction of expression of *PP2C* (a type C phosphatase), which attenuates late hook opening possibly as part of a mechanism that avoids an exaggerated response of hook opening to light stimulus once seedlings are de-etiolated (Sentandreu et al., 2011).

In darkness, COP1 directly ubiquitinates and targets to degradation in the 26S proteasome several transcription factors required for photomorphogenesis, including HY5 (LONG HYPOCOTYL 5) (see Box 1; Figure 4). BBX23 is also ubiquitinated by COP1 (Datta et al., 2008) and could therefore represent a point of convergence between COP1 and PIFs. Mutations in transcription factors genes downstream of COP1 signaling like *hyh (hy5 homolog*) show impaired hook opening under far-red light, but *hfr1* (*long hypocotyl in far-red*) and *laf1 (long after far-red light)* mutants are affected in hypocotyl elongation but not in hook opening under far-red light (Fairchild et al., 2000; Ballesteros et al., 2001). These data support the idea that, even though the apical hook structure is a part of the hypocotyl, hook formation and hypocotyl straight growth are two processes regulated independently downstream of COP1. Also, COP1 is able to regulate the amount of EIN3/EIL1 proteins during photo-oxidative damage (Zhong et al., 2009) and hypocotyl growth responses (Liang et al., 2012) playing crucial roles in ethylene signaling. It would be interesting to determine if COP1 regulates EIN3/EIL1 during hook development.

Light decreases the level of gibberellins in Arabidopsis (Ait-Ali et al., 1999; Achard et al., 2007; Alabadí et al., 2008) pea (Weller et al., 2009) and rice (Hirose et al., 2012) (Figures 3, 4). Blue light acting through cry1 reduces the expression of genes involved in gibberellin biosynthesis and the levels of this hormone (Folta et al., 2003; Foo et al., 2006). In addition, the pea *HY5* gene induces the expression of gibberellins-2-oxidase, the major gibberellin catabolic enzyme in plants, resulting in negatively regulation of gibberellin pathways (Weller et al., 2009). Additionally, phyA and phyB activities increase the accumulation of DELLA proteins (Achard et al., 2007). As mentioned before, EIN3/EIL1 are direct targets of DELLA proteins, which inhibits their function affecting their downstream targets elements. So, light might reduced EIN3/EIL1 availability through its dual effects on PIFs and on DELLA proteins (Figure 3). It has been shown that gibberellin signaling deficiencies in the *cop1* mutant of several species (Alabadí et al., 2008; Weller et al., 2009; Tanaka et al., 2011) suggesting that COP1 might be necessary for establishing gibberellins levels in darkness for hook formation.

In the hooks, light also affects the expression and localization of the auxin transporter ABCB19. ABCB19 localizes to the inner side of etiolated hooks but its presence fades away during hook opening under blue light (Wu et al., 2010) (Figure 3). Red or blue light perceived by phyA, phyB, and/or cry1 reduce ABCB19 protein levels in the upper section of the hypocotyls (Nagashima et al., 2008). There might be additional effects of light on auxin transporters during hook opening as at least during phototropic responses, light causes changes in the subcellular distribution of selected PIN carriers in the hypocotyls (Blakeslee et al., 2004; Ding et al., 2011). PIN1 in the hypocotyls and PIN1 and PIN2 intracellular distribution and abundance in the roots are light-regulated and controlled by COP1 (Sassi et al., 2012).

In summary, COP1 and PIFs are direct key targets of light signaling to control hook opening. PIFs operate downstream of gibberellins and affect auxin transport and ethylene synthesis. EIN3/EIL1 are indirect targets of light signaling, through the PIFs and DELLA pathways, operate downstream gibberellins and ethylene and affect auxin transport, synthesis and signaling. The connections between COP1 and the hormonal network that underlies the formation of the hook in darkness are not firmly established.

## Concluding remarks

The coordinated actions of gibberellins, ethylene and brassinosteroids control asymmetric auxin distribution to allow the correct development of the hook in darkness. This control occurs at multiple levels, including synthesis, distribution by the action of specific transporters and abundance of transcription factors involved in the control of auxin-responsive genes. PIFs and EIN3/EIL1 are key connectors of gibberellins and ethylene pathways with auxin signaling in the hooks. The repressors of photomorphogenesis PIFs and COP1 are indispensable for hook development in darkness and the activation of phytochromes and cryptochromes by light negatively regulates the activity of PIFs and COP1, leading to the opening of the hook.

Considerable progress has been made in recent years in the elucidation of the signaling network that controls apical hook formation in darkness; however there still are several gaps concerning how light-regulated proteins modulate hook formation and triggers hook opening. Learning the dynamics of the hormonal networks in response to light is a challenge for future research.

### Conflict of interest statement

The authors declare that the research was conducted in the absence of any commercial or financial relationships that could be construed as a potential conflict of interest.
